# 

**Published:** 1896-04

**Authors:** 


### CONTENTS—APRIL, 1896.—VOL. XI., NO. 10.

ORIGINAL COMMUNICATIONS—
Mental Therapy. Matthew M. Smith, M.D., Austin, Texas . . . . 533
Extroversion of the Bladder. A. E. Spohn, M.D., Corpus Christi, Tex. 540
Resection of Humerus. W. W. Pugh, M.D., Hearne, Texas . . . . 545

CORRESPONDENCE—
Castration for Crime not Justifiable nor Effective. Dr. DeCausey . . 547

CURRENT MEDICAL LITERATURE—
Department of Therapeutics. Edited by David Cerna, M.D., Galveston 549

SOCIETY NOTES—
Texas State Medical Association . . . . . . . . . . 552
Richmond Academy of Medicine and Surgery . . . . . . . . 559
To the Medical Profession of Texas . . . . . . . . . 562
Medico-Legal Society of Chicago . . . . . . . . . . 564
American Medical Association . . . . . . . . . . 565
Johnson County Medical Society . . . . . . . . . . 566

ABSTRACTS AND SELECTIONS—
The Surgical Treatment of Retro-Deviations of the Uterus . . . . . 566
Purification of Drinking Water by Filtration . . . . . . . 568
Death to Small Publishers . . . . . . . . . . . 570
The Medical Journal is the True Post-Graduate Instructor . . . . . 572
Rules for the Surgeon . . . . . . . . . . . 573
"My Attention," etc. . . . . . . . . . . . . 574

EDITORIAL—
An Alleged New Treatment for Tuberculosis . . . . . . . 575
The Committee . . . . . . . . . . . . 580
A Warning to Doctors . . . . . . . . . . . 581

MEDICAL NEWS AND MISCELLANY—
Many Interesting Paragraphs . . . . . . . . . . 582
Circular to Physicians . . . . . . . . . . . 590

PUBLISHERS' NOTES . . . . . . . . . . . . 591

